# Long-Term Toxicity of 50-nm and 1-μm Surface-Charged Polystyrene Microbeads in the Brine Shrimp *Artemia parthenogenetica* and Role of Food Availability

**DOI:** 10.3390/toxics11040356

**Published:** 2023-04-09

**Authors:** Yu Shen, Mingxing Zhang, Zhaochuan Li, Shuo Cao, Yadi Lou, Yi Cong, Fei Jin, Ying Wang

**Affiliations:** 1 College of Marine Science and Environment, Dalian Ocean University, Dalian 116023, China; sydlou@163.com; 2 Key Laboratory for Ecological Environment in Coastal Areas, National Marine Environmental Monitoring Center, Ministry of Ecology and Environment, Dalian 116023, China; 3 Marine Debris and Microplastic Research Center, Department of Marine Chemistry, National Marine Environmental Monitoring Center, Dalian 116023, China; 4 College of Marine Ecology and Environment, Shanghai Ocean University, Shanghai 201306, China

**Keywords:** microplastics, nanoplastics, amnio-modified polystyrene, chronic toxicity, food supply, uptake, zooplankton

## Abstract

Micro and nanoplastics (MNPs) as emerging contaminants have become a global environmental issue due to their small size and high bioavailability. However, very little information is available regarding their impact on zooplankton, especially when food availability is a limiting factor. Therefore, the present study aims at evaluating the long-term effects of two different sizes (50 nm and 1 μm) of amnio-modified polystyrene (PS-NH_2_) particles on brine shrimp, *Artemia parthenogenetica,* by providing different levels of food (microalgae) supply. Larvae were exposed to three environmentally relevant concentrations (5.5, 55, and 550 μg/L) of MNPs over a 14-days of exposure with two food levels, high (3 × 10^5^~1 × 10^7^ cells/mL), and low (1 × 10^5^ cells/mL) food conditions. When exposed to high food levels, the survival, growth, and development of *A. parthenogenetica* were not negatively affected at the studied exposure concentrations. By comparison, when exposed to a low food level, a U shape trend was observed for the three measured effects (survival rate, body length, and instar). Significant interactions between food level and exposure concentration were found for all three measured effects (three-way ANOVA, *p* < 0.05). The activities of additives extracted from 50 nm PS-NH_2_ suspensions were below toxic levels, while those from 1-μm PS-NH_2_ showed an impact on *artemia* growth and development. Our results demonstrate the long-term risks posed by MNPs when zooplankton have low levels of food intake.

## 1. Introduction

Microplastics (MPs, 1 μm~5 mm in size) and nanoplastics (NPs, <1 μm in size), as typical emerging contaminants, have caused global environmental concerns with the increase in plastic production [1]. The ecological risks that larger plastics pose to marine organisms have been widely reported, yet there are still knowledge gaps regarding the adverse impacts of micro and nanoplastics (MNPs) caused by their complexity in size, shape and nature. Microbeads, as a typical dominant MNP, can enter the marine environment via direct release through their use in personal care products [2]. Ingestion of MNPs by marine organisms have been reported due to their high bioavailability [3,4,5,6,7] and can cause several detrimental effects, including intestinal injuries, oxidative stress, triggering inflammation, and impacting fitness [1].

Zooplankton provide a key link between primary producers and higher trophic levels and plays an important role in the transportation of energy transfer and aquatic pollutants across the marine food web [8]. Different types of MNPs, can be ingested by zooplankton, which has been confirmed both through field studies and laboratorial studies [9,10]. The ingestion of MNPs by zooplankton causes a series of adverse effects on feeding behavior, growth, survival, development and reproduction [4,11,12,13,14].

Some factors, including particle size and exposed concentrations of MNPs, are important for explaining plastic particle ingestion and associated toxic effects on zooplankton. Previous results indicated that *Artemia parthenogenetica* larvae have a varying capacity to consume 10 μm polystyrene microbeads that is dependent on microplastic exposure concentration, exposure time, and the availability of food [11]. Another laboratorial study has also shown that 6 μm microbeads were more effectively ingested from the rotifer *Brachionus koreanus* than 0.05 or 0.5 μm microbeads and then toxicity of polystyrene microbeads, was size-dependent [15]. Moreover, the interference of food shortages with the adverse effects of MPs has also been reported. For example, uptake of polyethylene MPs (diameter, 10~22 μm) significantly reduced the net reproductive rate and intrinsic rate on the freshwater rotifer *Brachionus calycifloru* by providing lower food densities [16]. However, little is known about the long-term impact of MNPs on zooplankton with the consideration of several factors, including their size, exposure concentrations, and food supply.

In the current study, the brine shrimp (*A. parthenogenetica*), a filter-feeding organism, was chosen as the model species due to its wide use in ecotoxicological studies [17]. PS-NH_2_ microbeads, the majority adopted as model NMPs in toxicological studies, were chosen with the consideration of their high toxicological risk for humans and aquatic animals due to their similar molecular structure to proteins, which allow them to pass more easily through cell membrane [18]. We hypothesized that the stress from a low food supply could increase the adverse effects on survival, growth, and development caused by different sizes of PS-NH_2_ microbeads. To test the hypothesis, the long-term impacts of 50 nm and 1 μm PS-NH_2_ microbeads at three environmentally relevant concentrations (5.5, 55, and 550 μg/L) on *A. parthenogenetica* with a varying food supply over a 14-d exposure were investigated. In addition, impacts of the additives in formulations of PS-NH_2_ microbeads on *A. parthenogenetica* were considered throughout all experiments, and it is expected that there would be no significant toxicity due to its low concentration. This study will provide a better insight into the long-term toxicity of MNPs in zooplankton with the consideration of food supply.

## 2. Materials and Methods

### 2.1. Characterization and Preparation of PS-NH_2_

Two different sizes of non-fluorescently labelled amnio-modified polystyrene (PS-NH_2_) microbeads (100 g/L; Bangs Laboratories Inc., Fishers, IN, USA) with diameters of 50 nm and 1 μm were used for toxicity tests. 1 μm and 50 nm non-fluorescent PS-NH_2_ (100 g/L) were preserved in 322.35 and 48.33 mg/L of sodium azide (NaN_3_) solution, respectively. The 1 μm fluorescently labelled PS-NH_2_ microbeads (25 g/L; Merck, Darmstadt, Germany) with an excitation of 470 nm/emission of 505 nm were used for ingestion tests. Prior to each experiment, the stock solutions of 50 nm and 1 μm non-fluorescent PS-NH_2_ were prepared as 110 mg/L in ultrapure water (18.2 MΩ · cm) for physical characterization and preparation of test solutions and were kept at 4 °C until use.

Non-fluorescently labelled PS-NH_2_ with diameters of 50 nm and 1 μm were characterized using dynamic light scattering (DLS) (Malvern Zetasizer Nano ZS90, Malvern Instruments Ltd., Malvern, UK) and a transmission electron microscope (TEM) (HT7800, Hitachi, Tokyo, Japan). The hydrodynamic diameter (Z-average), polydispersity index (PDI), and zeta potential were measured as key parameters describing PS-NH_2_ behavior in ultrapure water. Measurements were carried out in triplicate. The primary morphology and particle size of PS-NH_2_ were identified using TEM at 80 kV.

An aliquot of the stock solution (110 mg/L) was sonicated for 5 min at 100 Hz using a CNC ultrasonic cleaner (KQ-500DB, Kun Shan Ultrasonic Instruments Co., Kunshan city, China) and diluted in glass crystallizing dishes with a 6 mm diameter containing filtered artificial seawater (FASW, salinity 30 ± 1, pH 8.0 ± 0.1, dissolved oxygen ~8.4 mg/L) and food (microalgae) to achieve the test solutions with a final concentration of 5.5, 55, and 550 μg/L for both toxicity and ingestion experiments. A recent field study in the Dutch Wadden Sea indicated that the mean concentration of polystyrene nanoplastics throughout the water column was 4.2 μg/L (range: 0.1–6.5 μg/L) [19]. Thus, the lowest test concentration, 5.5 μg/L, used in this study is environmentally relevant.

To exclude the effects of additives in commercial formulations on the toxicity of PS-NH_2_, the filtrate of PS-NH_2_ without plastic particles at the highest test concentration (550 μg/L) was obtained. The treatment process was briefly described below. Firstly, to remove plastic particles from the formulations, aliquots of the stock solution (110 mg/L) were centrifuged at 16,000× *g* for 2 h at 4 °C. Then, 10 mL of supernatant was transferred into syringes equipped with a membrane filter (0.02 μm, Whatman, UK). The filtrate after filtration was achieved in a 25 mL glass vial stored at 4 °C until use and diluted by FASW in glass crystallizing dishes for the toxicity test of 550 μg/L of PS-NH_2_ additives.

### 2.2. Experimental Organisms

Dried cysts of *A. parthenogenetica* obtained from a commercial source (Haitian Rongcheng Technology Co., Ltd., Tianjin, China) were stored at −20 °C prior to use. The hatching condition was based on our previous study [11]. Newly hatched nauplii (<24 h) were collected and cultured at 25 ± 1 °C under a 15 h light/9 h dark cycle for experiments.

### 2.3. Experimental Design

Two experiments were carried out to investigate the long-term toxicity of PS-NH_2_ with diameters of 50 nm and 1 μm to *A. parthenogenetica* (Exp. I) and PS-NH_2_ ingestion by *A. parthenogenetica* (Exp. II), respectively. The influencing factors in Exp. I mainly included the levels of food provided, exposure concentrations of PS-NH_2,_ and PS-NH_2_ size. Fresh marine diatoms *Chaetoceros muelleri,* were provided as a food source during the 14-d exposure period. The uptake of fluorescent PS-NH_2_ particles by *Artemia* was observed in Exp. II.

In Exp. I, chronic toxicity effects such as survival, growth, and development were investigated at two different food levels. The experimental groups consisted of FASW (control group), additive (filtrate of 550 μg/L PS-NH_2_), and PS-NH_2_ (5.5, 55, and 550 μg/L) with diameters of 50 nm and 1 μm. Two different food levels were chosen in Exp. Ia (1 × 10^5^ cells/mL during the whole 14-day exposure) and Exp. Ib (3 × 10^5^ cells/mL (days 1–4), 1.8 × 10^6^ cells/mL (days 5–8), and 1 × 10^7^ cells/mL (days 9–14). These algal densities were set mainly based on a previous study that focused on the influence of food resources on the toxicity of microplastics in the rotifer *Brachionus calyciflorus* [16].

Ten healthy *A. parthenogenetica* nauplii (instar II) were chosen and exposed to 20 mL of the test solutions mentioned above in glass crystallizing dishes. The exposure experiments were conducted under the same conditions as organism culture. The test solutions were renewed completely every other day during the exposure. Each exposure experiment was conducted with three replicates. After the end of exposure, *A. parthenogenetica* was rinsed three times with FASW to remove plastic particles or microalgae adhering to their body surface. The survival of *A. parthenogenetica* was observed daily, and dead animals were picked out of the crystallizing dishes immediately under the stereomicroscope after observation. At the end of the exposure, the number of survivors was recorded and the survival rate (%) was calculated. Moreover, the developmental stages were observed, and body length was measured under the stereomicroscope at the end of exposure. The specific measurement method and judging criteria were provided in our previous study [17].

In Exp. II, we conducted a 14-d exposure by using fluorescently labelled PS-NH_2_ with a diameter of 1 μm to verify that *A. parthenogenetica* had the capacity to ingest plastic particles. The number of exposed animals, microplastic exposure concentrations, exposure conditions, and food level are the same as those in Exp. Ib. At the end of exposure, *A. parthenogenetica* and their fecal pellets were picked out, transferred to clean glass scintillation vials, and fixed in 1 mL 2.5% buffered glutaraldehyde for more than 2 h. Then they were photographed under a fluorescent stereomicroscope (Leica M205FA, Wetzlar, Germany). The number of surviving individuals with ingested PS-NH_2_ particles was recorded, and the percentage of PS-NH_2_ uptake (%) was calculated by dividing the number of ingested individuals by the total number of exposed animals for each replicate.

### 2.4. Statistical Analyses

Statistical analyses were conducted using the SPSS 24.0 software package. Data were tested for normality distribution and homogeneity of variance using a Kolmogorov-Smirnov test and Levene’s test, respectively. The toxicity effects of exposure concentration, size, and food level of PS-NH_2_ and their interactions on survival, growth, and development in Exp. I was analyzed by three-way analysis of variance (ANOVA), and different treatments were compared by Bonferroni multiple comparisons for the analysis of significant differences. A one-way ANOVA with Dunnett’s and Tukey’s post hoc tests were used to compare the effects of additives in Exp. I and the percentage of PS-NH_2_ uptake in Exp. II, respectively. Data are presented as mean ± standard deviations (SD), and statistical significance was considered *p* < 0.05.

## 3. Results

### 3.1. PS-NH_2_ Characterization

**Figure 1:**
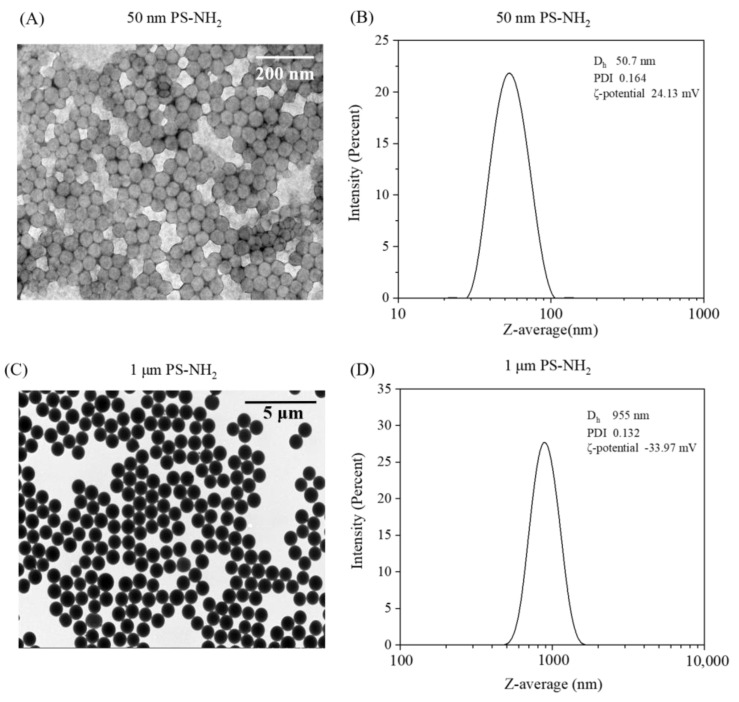
TEM images (**A**,**C**) and DLS measurements (**B**,**D**) of PS-NH_2_ MNPs in ultrapure water. Dh, PDI, and ζ-potential represent hydrodynamic diameter (nm), polydispersity index (PDI), and zeta potential (mV), respectively.

The physio-chemical properties of the 50 nm and 1 μm PS-NH_2_ MNPs are shown in Figure 1. TEM images (Figure 1A,C) revealed that PS-NH_2_ had a fairly spherical shape, and the size was close with the results obtained by DLS. Their sizes for the 50 nm and 1 μm PS-NH_2_ MNPs analyzed by DLS were 50.7 ± 0.8 and 955.0 ± 24.194 nm (Figure 1B,D), respectively. The zeta-potential values of 50 nm PS-NH_2_ and 1 μm PS-NH_2_ were +24.13 ± 0.660 and −33.97 ± 0.447 mV, respectively.

### 3.2. Long-Term Effects of 50-nm and 1-μm PS-NH_2_ on Survival, Growth and Development of Brine Shrimp by Providing Low Food Level

**Figure 2:**
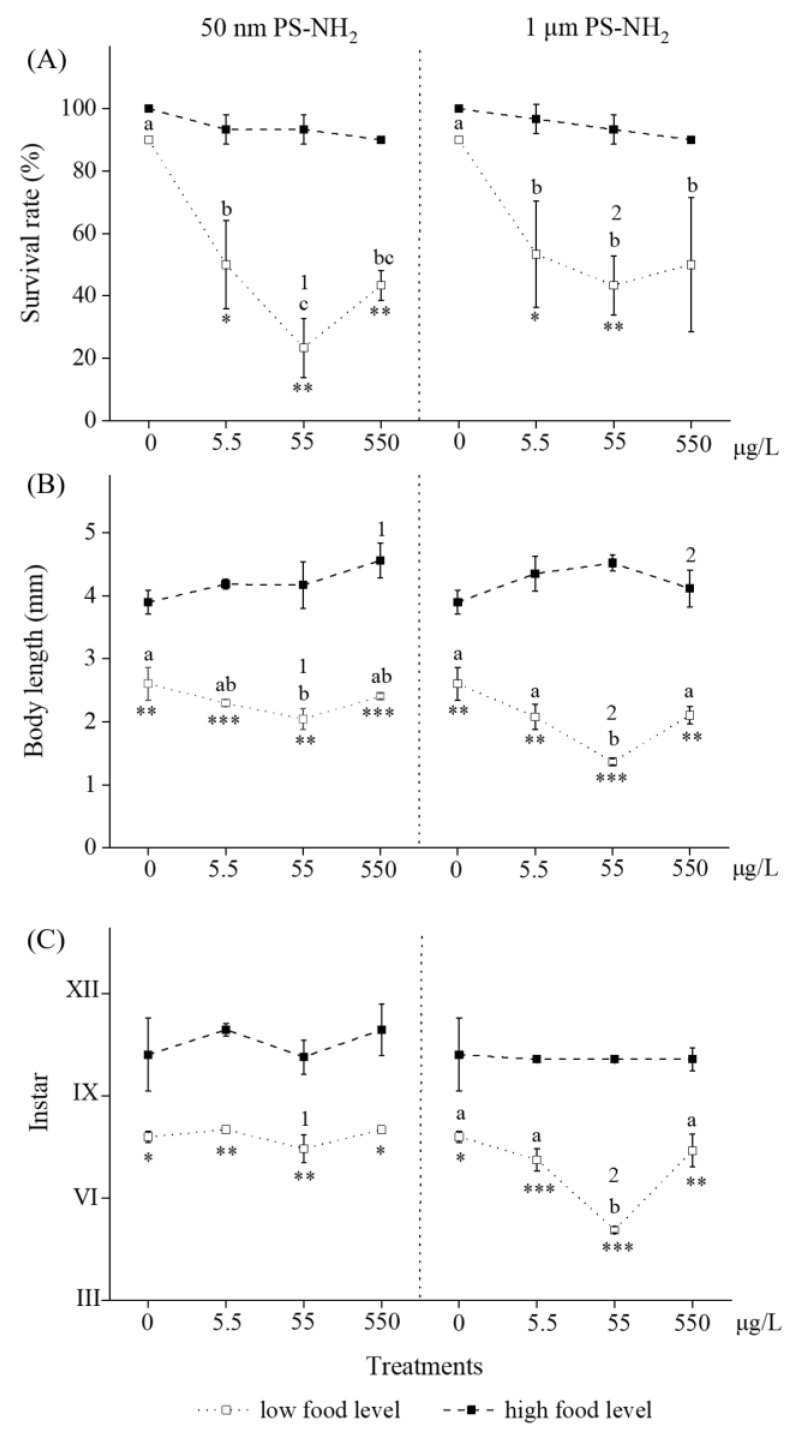
Survival rate (%) (**A**), body length (mm) (**B**), and instar (**C**) for *A. parthenogenetica* exposed to 50 nm and 1 μm PS-NH_2_ for 14 days. Data were analyzed by three-way ANOVA, and different treatments were compared by Bonferroni multiple comparisons for the analysis of significant differences. Error bars represent means values ± SD. Different letters/numbers indicate significant differences between exposure groups with different concentrations/sizes (*p* < 0.05), while differences between treatments by providing low and high food levels are shown as * (*p* < 0.05), ** (*p* < 0.01), and *** (*p* < 0.001).

With regard to survival rate, individuals upon exposure to 50 nm/1 μm PS-NH_2_ by providing low food levels found that the interaction between exposure concentration and size was not significant (*F*_(3,16)_ = 0.532, *p* > 0.05). For the concentration effect, there was a significant effect on survival rate among treatments of exposure concentrations (0, 5.5, 55, and 550 μg/L) (*F*_(3,16)_ = 16.378, *p* < 0.05). The treatments showed a decreasing trend with the increase of exposure concentrations for two different sizes of PS-NH_2_ (Figure 2A). The 55 μg/L experimental group for 50-nm PS-NH_2_ had the lowest survival rate (23.33 ± 9.43%), slightly lower than that (43.33 ± 4.71%) in the 550 µg/L experimental group, and there was no significant difference between the two groups (*p* > 0.05). Similar results on the survival rate influenced by exposure concentration for 1 μm PS-NH_2_ were also obtained. For the size effect, there was no significant effect on the survival rate between treatments of 50 nm and 1 μm PS-NH_2_ (*F*_(3,16)_ = 1.558, *p* > 0.05).

With regard to body length, individuals upon exposure to 50 nm/1 μm PS-NH_2_ by providing low food levels found that the interaction between exposure concentration and size was not significant (*F*_(3,16)_ = 2.825, *p* > 0.05). For the concentration effect, there was a significant effect on body length between treatments of 50 nm and 1 μm PS-NH_2_ (*F*_(3,16)_ = 19.264, *p* < 0.05). The treatments showed a slightly decreasing trend with the increase of exposure concentrations for two different sizes of PS-NH_2_ (Figure 2B). The 55 μg/L experimental group for 50 nm and 1 μm PS-NH_2_ had the lowest body length, with respective values of 2.046 ± 0.168 mm and 1.363 ± 0.050 mm, while the body length of their control group was 2.604 ± 0.258 mm. For the size effect, there was a significant effect on body length between treatments of 50-nm and 1-μm PS-NH_2_ (*F*_(3,16)_ = 12.641, *p* < 0.05).

With regard to instar, individuals upon exposure to 50 nm/1 μm PS-NH_2_ by providing low food levels showed an interaction between exposure concentration and size that was significant (*F*_(3,16)_ = 14.257, *p* < 0.05). For the concentration effect, there was a significant effect on survival rate between treatments of 50 nm and 1 μm PS-NH_2_ (*F*_(3,16)_ = 29.158, *p* < 0.05). The treatments also showed a slightly decreasing trend with the increase of exposure concentrations for two different sizes of PS-NH_2_ (Figure 2B). The 55 μg/L experimental group for 50 nm and 1 μm PS-NH_2_ had the lowest instar, with respective values of 7.44 ± 0.42 and 5.07 ± 0.09, while the instar of their control groups was 7.80 ± 0.16. For the size effect, there was a significant effect on instar between treatments of 50 nm and 1 μm PS-NH_2_ (*F*_(3,16)_ = 52.323, *p* < 0.05).

### 3.3. Long-Term Effects of 50-nm and 1-μm PS-NH_2_ on Survival, Growth and Development of Brine Shrimp by Providing High Food Level

When providing a high food level, no significant adverse effects on survival, growth, or development of brine shrimp upon exposures to 50 nm/1 μm PS-NH_2_ were shown relative to their respective controls (Figure 2, *p* > 0.05). The interaction between exposure concentration and size was not significant (all *p* > 0.05).

### 3.4. Effects of Food Level on Long-Term Toxicity of Selected MNPs

On the whole, compared to low food levels, no long-term adverse effects were observed on the survival, growth, and development of the brine shrimp upon exposure to 50 nm/1 μm PS-NH_2_ for 14 days, while high food levels were provided (Figure 2, *p* < 0.05). Food level is the most important factor affecting the final toxicity of MNPs in this study (Table 1, three-way ANOVA, all *p* < 0.05).

When measuring the survival rate for individuals upon exposure to 50 nm and 1 μm PS-NH_2,_ a significant interaction between exposure concentration and food level was found (*F*_(3,32)_ = 11.310, *p* < 0.05). For 50 nm PS-NH_2_, the survival rates of the three experimental groups (5.5, 55, and 550 μg/L) at high food levels were significantly higher than those at low food levels (*p* < 0.05) (Figure 2A), although the control survival rate at low food levels was not significantly different from that at high food levels. Specifically, the significantly reduced survival rate at low food level (23.33 ± 9.43%) in the 55 μg/L experimental group was found compared to that at high food level (93.33 ± 4.71%) (*p* < 0.01). Similar results were also obtained for the 1 μm PS-NH_2_ treatment.

When measuring body length for individuals upon exposure to 50 nm and 1 μm PS-NH_2_, significant interactions between food level and exposure concentration/size were found (*F*_(3,32)_ = 14.095 and *F*_(1,32)_ = 4.686, both *p* < 0.05). For 50 nm PS-NH_2_, the body length of all the experimental groups (0, 5.5, 55, and 550 μg/L) at a high food level was significantly higher than that at a low food level (*p* < 0.05) (Figure 2B). Specifically, the significantly reduced body length at low food level (2.046 ± 0.168 mm) in the 55 μg/L experimental group was found compared to that at high food level (4.167 ± 0.369 mm) (*p* < 0.01). Similar phenomena were found for individuals upon exposure to the 1 μm PS-NH_2_ group.

**Figure 3:**
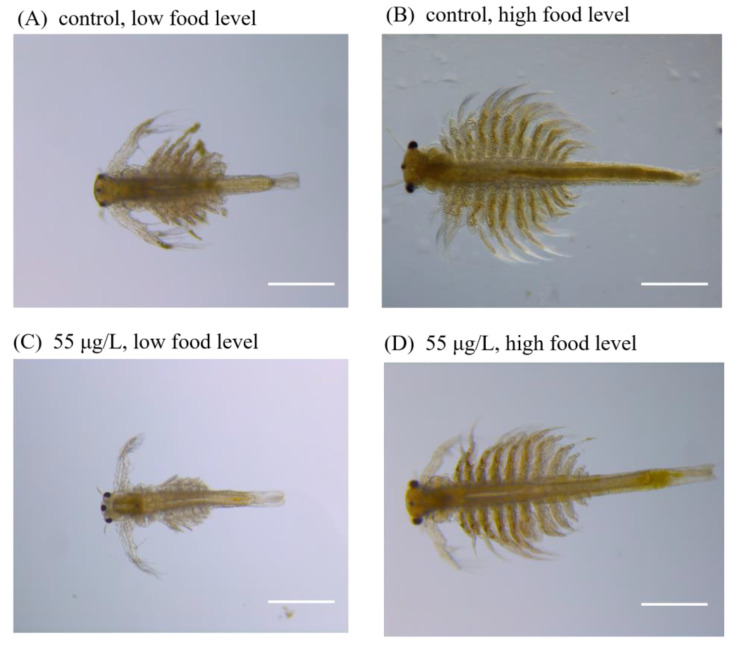
Typical images of *A. parthenogenetica* at the end of 14 d exposure in Exp. Ia (low food level) and Exp. Ib (high food level) for the control groups (**A**,**B**) and 1 μm PS-NH_2_ (55 μg/L) groups (**C**,**D**). The scale bar is 500 μm.

When measuring instar for individuals upon exposure to 50 nm and 1 μm PS-NH_2,_ a significant interaction between exposure concentration and food level was found (*F*_(3,32)_ = 3.088, *p* < 0.05). For 50 nm PS-NH_2_, the instars of all the experimental groups (0, 5.5, 55, and 550 μg/L) at high food levels were significantly higher than those at low food levels (*p* < 0.05) (Figure 2C). Specifically, the significantly decreased instar at low food level (7.44 ± 0.41) in the 55 μg/L experimental group, was found compared to that at high food level (10.10 ± 0.09) (*p* < 0.01). Similar phenomena were found for individuals upon exposure to the 1 μm PS-NH_2_ group. The typical images of individuals exposed to the 55 µg/L PS-NH_2_ are shown in Figure 3. 

### 3.5. Effects of Additives in Plastic Commercial Formulations on Toxicity

**Figure 4:**
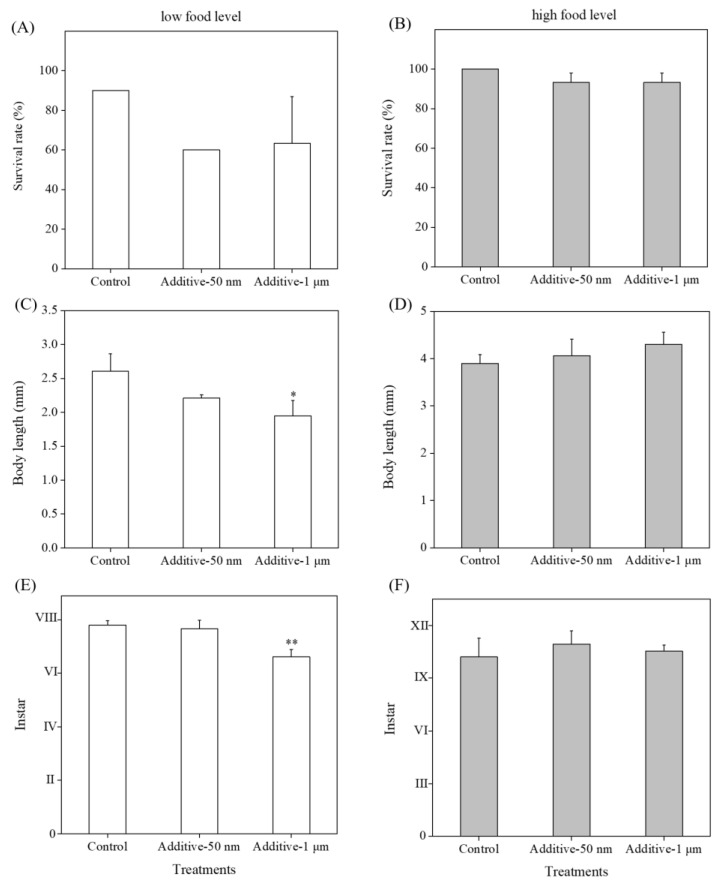
Impacts of additives extracted from 50 nm and 1 μm PS-NH_2_ on survival rate (%), body length (mm) and instar for *A. parthenogenetica* upon 14 day exposure to low food levels (**A**,**C**,**E**) and high food levels (**B**,**D**,**F**). Column bars represent means values ± SD and differences between exposure groups and controls are shown as * (*p* < 0.05) and ** (*p* < 0.01) using one-way ANOVA with Dunnett’s post hoc test.

The adverse effects of additives in commercial formulations of MNPs on brine shrimp were investigated in this study. The additives mainly containing NaN_3_ (7.09 μg/L) in the 1 μm PS-NH_2_ group (550 μg/L) showed reduced body length and instar of the brine shrimp when providing a low food level (one-way ANOVA, *p* < 0.05, Figure 4C,E). In comparison, when providing a high food level, all endpoints related to survival, growth, and development were not significantly affected relative to their respective controls (*p* > 0.05, Figure 4B,D,F).

### 3.6. Microplastic Uptake by Brine Shrimp upon Exposures to 1 μm PS-NH_2_ When Providing High Food Level

**Figure 5:**
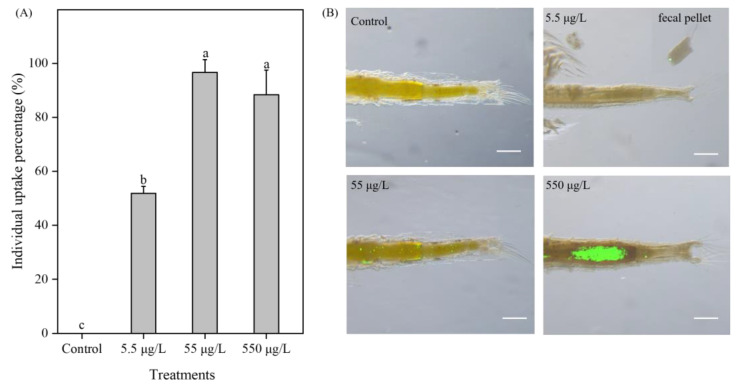
Uptake percentage for *A. parthenogenetica* exposed to the control and 1 μm PS-NH_2_ (5.5, 55, and 550 μg/L) groups for 14 days provided with high food levels (**A**) and images of guts and fecal pellets (**B**). Column bars represent means values ± SD and different letters indicate a significant difference between treatments (one-way ANOVA with Tukey’s post hoc test, *p* < 0.05). The scale bar is 20 μm.

Only uptake of PS-NH_2_ with the diameter of 1 μm was investigated because ingestion and egestion by *A. parthenogenetica* cannot be recognized under microscope for PS-NH_2_ with the diameter of 50 nm due to the relatively low exposure concentrations used in this study. The percentages of 1 μm PS-NH_2_ uptake by *Artemia* individuals exposed to the three treatment groups (5.5, 55, and 550 μg/L) in Exp. II were 51.85%, 96.67%, and 88.43%, respectively (Figure 5A). The percentage of PS-NH_2_ uptake for all exposed individuals in the 5.5 μg/L group was significantly (*p* < 0.05) lower than the 55 μg/L and 550 μg/L groups (Figure 5A). The fluorescent labelled 1 μm PS-NH_2_ particles were observed in the guts and fecal pellets of *A. parthenogenetica* after a 14-day exposure to PS-NH_2_ solutions (5.5, 55, and 550 μg/L) (Figure 5B). *A. parthenogenetica* have different capacities to ingest PS-NH_2_ when exposed to 1 μm PS-NH_2_ solutions with different concentrations (Figure 5B). It can be found that the observed fluorescent intensity increased with the increase in the PS-NH_2_ exposure concentration.

## 4. Discussion

The potential uptake and toxicity of microplastics and nanoplastics are of particular concern for marine organisms at lower trophic levels, such as zooplankton. Due to their small size, a wide range of marine zooplankton species can readily ingest them, which might further result in the transfer to higher trophic levels [20,21]. The factors that contribute to the bioavailability of MNPs to zooplankton relate to their physiochemical properties [1]. Therefore, recent toxicological studies have focused on the adverse impact of particle-specific properties (e.g., shape, size, concentration, chemical composition, surface charge) [11,22]. However, there are limited reports concerning the potential effects resulting from food shortages and their interactions with other factors, especially when multiple stressors co-exist in a real-life scenario. 

The concentration of the test MNPs turned out to be an important factor for explaining the toxic effect in *A. parthenogenetica*. We designed environmentally relevant concentrations of MNPs according to the extrapolation method [23]. It is difficult to know the real abundance of MNPs in the marine environment, especially for those with a diameter less than 1 μm due to the analytical challenges of reliable chemical analysis [24]. As described in our previous study, the environmentally relevant concentration of MNPs in small sizes can be extrapolated because their concentrations increase with the decrease of the sampling mesh sizes or their diameter [25]. Therefore, environmentally relevant concentrations of MNPs in surface seawater could be in the magnitude order of μg/L and three concentrations (5.5, 55, and 550 μg/L) were selected in this study. No traditional concentration-effect relationship was observed for MNPs (Figure 2) due to their special physical properties, rapid aggregation, and deposition in seawater [2]. Interestingly, a “U” shape for the toxic effect was seen in Figure 2 at low food levels. This phenomenon could be attributed to more aggregates of larger size forming at higher concentrations (550 μg/L) compared to lower concentrations (55 μg/L). Surface charge represents one of the main properties driving the behavior of MNPs in aquatic environments, including aggregation, mobility, and deposition [2]. Thus, only MNPs in proper size after aggregation in the test solutions are bioavailable and further exert toxicity effect in *A. parthenogenetica*. Further studies are necessary to take the size distribution of MNPs of the exposure media into account and quantify their internalized amount in vivo by uptake when interpreting the observed toxicity. 

Since food resources can affect the fitness of organisms, we hypothesized that the negative effects of nanoplastic and microplastic PS-NH_2_ particles would be more severe when providing a low food level compared to a high food level. Our experimental results verified the hypothesis: the long-term toxicity of 50 nm and 1 μm PS-NH_2_ in the brine shrimp *A. parthenogenetica* was strongly dependent on food availability. Specifically, we found that 50 nm and 1 μm PS-NH_2_ had a more severe adverse impact on survival, growth, and development by providing a low-level food supply, while no consequences were observed by providing a high-level food supply. In other words, more algal supply effectively decreased the negative effects of MNPs on *Artemia* in this study (Figure 2). A field study indicated that the dominant diatoms in southern Chinese coastal waters mainly included several species such as *Pseudonitzschia* spp., *Chaetoceros* spp., and *Synedra* spp., and the reported cell densities of *Pseudonitzschia* spp. varied from 0 to 6.16 × 10^3^ cells/mL, with an average of 4.75 × 10^2^ cells/mL [26]. Therefore, the food (diatom, *Chaetoceros muelleri*) supply, even at the low level in this study, was still much higher than the realistic abundance of microalgae, and the ecological risks posed to *Artemia* exposed to MNPs and under a real scenario could potentially exist. A similar study revealed that lower food levels increased the negative effects of microplastics on the benthic-dwelling invertebrate, *Chironomus riparius*, in the presence of 1 μm PS beads when food was a limiting factor [27]. Food availability is also crucial for the effects of 1 μm polystyrene beads on the nematode *Caenorhabditis elegans* in freshwater sediments [28].

The negative influences of MNPs in this study could be a result of reduced uptake of microalgae, especially when algal density is relatively low, because ingested MNPs might occupy the inner space of digestive tract, which reduces the uptake of microalgae [16]. We observed uptake of 1 μm PS-NH_2_ by *A. parthenogenetica* in the guts at the three exposure concentrations (5.5, 55, and 550 μg/L) and their egestion in feces (Figure 3). The ingestion capacity of zooplankton for microplastics and nanoplastics depends on the selective or nonselective feeding behavior of species and the properties of MNPs [3,4]. Thus, increased energy consumption led to reduced energy investment in growth and development. A study also indicated that MNPs can modulate the feeding capacity through such factors as grazing rate and prey selection and then alter the total energetic input in zooplankton [29]. The energy allocation, especially under low food levels, which may affect life-history traits including growth, development, and reproduction and ultimately individual fitness, remains to be further investigated. Moreover, the mechanical damage of ingested MNPs by *Artemia* might occur due to their transportation among tissues and organs after ingestion. Future studies applying histopathology and exploring cellular and tissue changes, are also welcome and could enable us to understand their toxicity mechanisms. Last but not least, accumulation of MNPs in *A. parthenogenetica*, a filter feeding animal, may affect their swimming behavior and the capacity of gathering algal food [16]. 

The usage of additives or preservatives in many commercially available formulations might affect the final toxicity results caused by MNPs themselves [30]. A simple in-situ method by using filtration has been developed to consider the toxic effects of additives in our previous study [25]. In this study, we adopted the same method to assess the adverse impact of the additive (sodium azide) in test solutions on *Artemia*. Only the body length and instar of individuals upon exposure to 550 μg/L of 1 μm PS-NH_2_ by providing low food levels were affected by the presence of 7.09 μg/L of additive (NaN_3_) (Figure 4C,E). Thus, the adverse effects on growth and development of *Artemia* for 1-μm PS-NH_2_ we achieved were induced by the co-presence of the additives and MPs. Similarly, previous results revealed that the dialyzed polystyrene nanoplastics did not cause mortality but significantly disrupted the swimming behavior of *Daphnia magna* [30]. Further studies are still needed to highlight the importance of considering the impacts of the suspension matrix of commercial particle formulations containing various preservatives or surfactants.

## 5. Conclusions

Overall, our results showed that a high-level supply of algal food effectively reduced the negative effects of the tested MNPs on survival, growth, and development, which implies food level (algal density) is the most important factor affecting their final toxicities at their environmentally relevant concentrations. The exposure concentrations of the test MNPs might be another important factor for explaining the toxic effects, and the interaction with food level was also observed. Further studies on the influence factors of their bioavailability and toxic effects at tissue and cellular levels should be explored for MNPs under environmentally realistic multi-stressors.

## Figures and Tables

**Table 1:** Three-way ANOVA summary on the effects of exposure concentrations (C) and size (S) of PS-NH_2_, as well as food level (F), on survival rates (%), body length (mm), and instar of *A. parthenogenetica*. Exposure concentrations of PS-NH_2_: 0, 5.5, 55, and 550 μg/L; food level: low food level, high food level; Size: 50 nm, 1 μm; MSE: mean squared error.

Factors	MSE	*F*	*p*
Survival rate			
C	0.233	*F*_(3,32)_ = 20.000	<0.001 *
F	1.841	*F*_(1,32)_ = 157.786	<0.001 *
S	0.021	*F*_(1,32)_ = 1.786	0.191
C *F	0.132	*F*_(3,32)_ = 11.310	<0.001 *
C *S	0.005	*F*_(3,32)_ = 0.452	0.717
F *S	0.013	*F*_(1,32)_ = 1.143	0.293
C *F *S	0.007	*F*_(3,32)_ = 0.571	0.638
Body length			
C	0.173	*F*_(3,32)_ = 2.657	0.065
F	49.066	*F*_(1,32)_ = 753.944	<0.001 *
S	0.239	*F*_(1,32)_ = 3.675	0.064
C *F	0.917	*F*_(3,32)_ = 14.095	<0.001 *
C *S	0.087	*F*_(3,32)_ = 1.341	0.279
F *S	0.305	*F*_(1,32)_ = 4.686	0.038
C *F *S	0.206	*F*_(3,32)_ = 3.170	0.038
Instar			
C	2.264	*F*_(3,32)_ = 6.272	0.002 *
F	107.786	*F*_(1,32)_ = 298.604	<0.001 *
S	6.017	*F*_(1,32)_ = 16.670	<0.001 *
C *F	1.115	*F*_(3,32)_ = 3.088	0.041 *
C *S	0.793	*F*_(3,32)_ = 2.198	0.107
F *S	0.800	*F*_(1,32)_ = 2.215	0.146
C *F *S	1.085	*F*_(3,32)_ = 3.006	0.045 *

Note: * indicates *p* < 0.05.

## Data Availability

Data are contained within the article.
